# Radio Frequency Energy Harvesting Technologies: A Comprehensive Review on Designing, Methodologies, and Potential Applications

**DOI:** 10.3390/s22114144

**Published:** 2022-05-30

**Authors:** Husam Hamid Ibrahim, Mandeep Jit Singh, Samir Salem Al-Bawri, Sura Khalil Ibrahim, Mohammad Tariqul Islam, Ahmed Alzamil, Md Shabiul Islam

**Affiliations:** 1 Department of Electrical, Electronic and Systems Engineering, Faculty of Engineering and Built Environment, Universiti Kebangsaan Malaysia (UKM), Bangi 43600, Selangor, Malaysia; hussampc93@gmail.com (H.H.I.); engsurkh.18@gmail.com (S.K.I.); 2 Space Science Centre, Institute of Climate Change, Universiti Kebangsaan Malaysia (UKM), Bangi 43600, Selangor, Malaysia; 3 Department of Electronics & Communication Engineering, Faculty of Engineering & Petroleum, Hadhramout University, Al-Mukalla 50512, Hadhramout, Yemen; 4 Electrical Engineering Department, College of Engineering, University of Ha’il, Ha’il 81481, Saudi Arabia; aa.alzamil@uoh.edu.sa; 5 Faculty of Engineering, Multimedia University, Persiaran Multimedia, Cyberjaya 63100, Selangor, Malaysia; shabiul.islam@mmu.edu.my

**Keywords:** radio frequency energy harvesting, antenna, rectenna, rectifier, impedance matching network, voltage multiplier

## Abstract

Radio frequency energy harvesting (RF-EH) is a potential technology via the generation of electromagnetic waves. This advanced technology offers the supply of wireless power that is applicable for battery-free devices, which makes it a prospective alternative energy source for future applications. In addition to the dynamic energy recharging of wireless devices and a wide range of environmentally friendly energy source options, the emergence of the RF-EH technology is advantageous in facilitating various applications that require quality of service. This review highlights the abundant source of RF-EH from the surroundings sources, including nearby mobile phones, Wi-Fi, wireless local area network, broadcast television signal or DTS, and FM/AM radio signals. In contrast, the energy is captured by a receiving antenna and rectified into a working direct current voltage. This review also summarizes the power of RF-EH technology, which would provide a guideline for developing RF-EH units. The energy harvesting circuits depend on cutting-edge electrical technology to achieve significant efficiency, given that they are built to perform with considerably small current and voltage. Hence, the review includes a thorough analysis and discussion of various RF designs and their pros and cons. Finally, the latest applications of RF-EH are presented.

## 1. Introduction

To date, radio frequency (RF) harvesters offer exceptional advantages over other sources of ambient solar energy, such as solar, acoustic, and mechanical vibration harvesters. The various notable characteristics of RF energy harvesting technology, including reliability, predictability, controllability, and the ability to simultaneously supply energy to different nodes, have made it the preferable option for certain areas or applications [1,2]. Interestingly, the marginal total energy harvested allows it to be applied in many applications that expect lower power consumption and contain many nodes [3]. The RF energy can be divided into ambient and intended RF [3]. The intended RF emits an RF signal from an RF transmitter directly to a specific area [4]. The advanced development of semiconductor technology and the fabrication process allow for the RF-EH concept realization. The conversion of energy in electromagnetic waves (EM) into valuable electrical energy in RF-EH facilitates the real application of continuous operating sensors [5]. On the other hand, however, the ambient radiofrequency energy is accessible over a broad range of frequency bands, such as LTE (750–800 MHz), DTV (550–600 MHz), UMTS (2150–2200 MHz), GSM-900 (850–910 MHz), GSM-1800 (1850–1900 MHz), Wi-Fi (2.4–2.45 GHz), band for television (TV) and radio applications (900 MHz–2 GHz), ISM (2.1–2.6 GHz), UWB (3.1–10.6 GHz), WLAN (3.1–4.4 GHz), HIPERLAN (5.1–5.3 GHz), and C-BAND (4.4–5 GHz) [6]. 

Increasing attention is being paid to energy-harvesting technologies that use ambient power sources, including heat, vibration, and electromagnetic waves. The self-sustaining “Zero-Power” standalone electronics have been developed in various energy harvesting systems, such as circuitries, topologies, and energy harvesting devices [7]. Currently, Wi-Fi, Wi-MAX, and GSM applications are more favored because of the services provided, including voice and video calls, short message service (SMS), high-speed data transfer, and broadly accessible internet access. These functional applications are utilized in mobile phones, laptops, and other portable devices that radiate plentiful amounts of RF energy, which can be harvested [8]. Harvesting RF energy is all about protecting RF energy from the radio environment and putting it to use in low-voltage electronic devices. Antennas-like patches with ultra-wideband properties or narrow-band antennas are required to detect radio frequency energy emitted by the radio environment. However, the utilization of the latter depends on the frequency bands that are going to be detected. For instance, an antenna with narrowband properties is required to detect GSM-900 frequencies. In addition, multiple-input-multiple-output systems are employed to improve the functional properties of wireless communication systems [9].

Meanwhile, the disposal of battery waste has become a significant issue in the last few years. Battery waste is mainly disposed of in landfills, which would lead to land pollution and the contamination of underground water due to the dissipation of harmful chemicals from the battery. Besides applying wireless power harvesting (WPH) technology to minimize the reliance on batteries, avoiding the use of batteries is the most effective solution to reduce the issue of battery waste, although it is not ideal [6]. In view of this, wireless RF power harvesting is considered a promising approach to replace or increase the lifespan of batteries. In fact, the ability of RF-EH via ambient RF energy provides an alternative method to reduce the cost of regular maintenance in terms of device improvement [10]. It has been explored [11,12,13,14,15] if radiofrequency energy can be used to replace batteries in low-power embedded systems, given the various energy sources available in the environment. Although the main perspective is to improve the lifespan of multipurpose electronic components, the RF-EH technology is feasible and distinguished through the required applications and its ability in philosophical design. The feasibility of the overall system dramatically relies on the incorporation of modules comprising microwave antennas, direct current (DC) booster, impedance matching network, power management techniques, and rectifying circuits. Therefore, the development of the RF-EH system is defined through critical trade-offs that researchers employ to achieve an optimal system in specific applications [16,17]. 

Despite that, the available incident energy in RF-EH is the least among other alternative sources of energy, such as wind and solar energy. The RF-EH technology possesses many unique advantages. Most importantly, the method performs in any location with a strong radio frequency signal, including areas with no sunlight or indoor spaces with specialized transmitters. This review highlighted the RF-EH technology that converts the energy in RF signal incident at the antenna into usable DC power. Following a brief introduction, the following is how the review paper is organized: Section 2 describes the RF-EH system. The RF-EH approaches are discussed in Section 3. RF Concepts and Principles are provided in Section 4, while a review of the power-harvesting evaluation metrics is described in Section 5. Section 6 focuses on the brief details of the radio frequency environments. Section 7 is the explanation of the RF-EH circuit. Section 8 describes the comparison of the existing RF-EH applications. The review finally ends with a concise conclusion.

## 2. RF-EH System 

The RF-EH system has received much popularity over recent years, as the method offers an alternative yet the sustainable approach to supply power to low-powered electronic systems [18,19]. Among the common low-powered electronic systems include the internet of things (IoT)-powered devices, wireless sensor networks (WSN), and smart metering systems. Moreover, RF-EH is a suitable alternative and promising approach to deliver energy to next-generation wireless networks [20]. The energy generated by powerful RF signals can be used to charge an RF-EH circuit’s energy storage unit. For instance, supercapacitors or rechargeable batteries are time-efficient compared to weak signals [21]. The use of energy harvesters to provide power for WSNs is a promising solution. Sensor devices operate on the energy that already exists in the environment, rather than relying on centralized power sources to charge their batteries. To power integrated circuits, a holding capacitor or supercapacitor is used to keep the direct current voltage (DC voltage) that is used to power them [22]. The different output of the RF power presents a considerable setback in the configuration of RF-EHs since the harvester performance, including the efficiency, is proportional to the power input and the RF frequency source as a result of the impedance matching and the turn-on voltage of the RF-EH, respectively [22]. 

While the design methodology to achieve optimal RF-EH performance has been developed [23], the harvester must be capable of monitoring the frequency and the power level of the operating RF energy to consistently achieve optimum efficiency. In comparison to wireless power transmission (WPT), the nonreliance of power from the operator of the harvester is the key advantage of RF-EH, which constitutes a “free” energy source. Nevertheless, RF-EH is regarded as the most complex energy source due to the varying frequency over the available harvestable power, location, time, energy source distance, and environmental factors. Furthermore, RF-EH has a low power density, particularly in comparison to other renewable energy sources [24]. Despite these limitations, RF-EH faces only minor density problems, particularly the sub-microwatt state (for example, a cellular GSM base station that generated 0.1 μW/cm^2^ [25]), significantly different from other ambient energy sources from the sun, through motion, or electrochemical reaction. The small RF density can only be used in ultrapowered devices under constant operation (non-duty-cycled) or low-powered applications, such as duty-cycled operation and low-powered wireless sensors in delay-limited devices, given that an adequate amount of RF energy must be harvested prior to the operation of the system [25].

**Figure 1:**
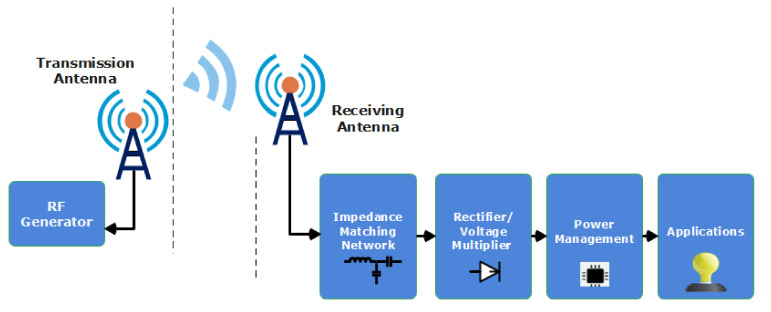
An RF energy harvesting systems conceptual block diagram.

A typical RF-EH circuit comprises a rectifier, a voltage multiplier, an antenna, and a device for energy storage. The most vital part of the RF-EH circuit is the rectifier, which significantly influences the system’s efficiency. The antenna serves as a transducer to convert the strength of an electric field into a voltage difference, or vice versa. The rectifier, on the other hand, converts RF power to DC power. The voltage multiplier produces a higher output DC voltage level when the sensor or energy storage device is activated. Once the energy has been harvested, batteries or supercapacitors are used to store it. It is critical to measure and investigate the power density of EM fields in the ambient environment before designing an RF-EH circuit. The electromagnetic spectrum has been measured in many countries [26,27] since the preferred frequency band, or bands for radiofrequency energy-harvesting circuits, are the most powerful. The structure of the RF-EH system is illustrated in Figure 1 [28]. 

Wireless communication systems’ RF signals would be the best suitable renewable energy source. Energy sources are based on vibration, light, and heat, and are not continuously present everywhere. In contrast, electromagnetic energy occurs in varying magnetic fields surrounding the alternating current power lines or the emission of radio waves from nearby transmitters. The EH application devices are categorized as near-field and far-field [29,30]. 

## 3. RF-EH Techniques 

Contrary to other energy-harvesting sources, such as wind, solar, and vibrations, the RF-EH is characterized as follows:

(a) Ability to regulate and provide constant energy transfer over a long distance.
(b) The harvested energy is relatively stable and predictable for long-term performance to the fixed distance in an RF-EHN setup.
(c) The different locations of network nodes exhibit a substantial difference in the RF-RH since the total RF-EH relies on how far the dedicated RF source is from the ambient RF source.

### 3.1. RF-EH from Dedicated RF Sources

The two options are dedicated transfer, which necessitates high power values, and ambient energy harvesting, which necessitates low power values. A circuit that harvests RF energy from a dedicated source over a short distance is expected to generate power levels in the range of 50 nW/cm^2^. One example is an RFID chip that is powered by an RFID reader. Because of the dedicated power supply, embedded devices can recharge their batteries [31]. Path loss, energy dissipation, shadowing, and fading are some of the drawbacks of RFH, whereas RFET has a number of promising directions and an advantage over nonradiative wireless energy transfer in terms of relaxed coupling/alignment specifications. Reception sensitivity and maximum radiation limits are all factors in the problem, as is a dramatic reduction in RF conversion efficiency at low receive powers. Additionally, the information reception sensitivity in RFH (typically −60 dBm in data reception vs. −10 dBm in RFH) is several orders of magnitude higher than in wireless data transfer. Due to the current state of the device and RF circuit technology, some applications may not be feasible. This two-hop decode-and-forward relay mode may not be able to work with conventional internode distances (a few tens of meters) because the current achievable energy transfer range is only a few millimeters [32].

### 3.2. RF-EH from Ambient RF Sources

This source type is further subdivided into two categories:

- Static sources, even though they are stable-power transmitters, are not simplified; the sensor device’s power is supplied by modulating the signal (for instance, by modulating the frequency and transmitted power). Ambient sources, including broadcast radio, mobile base stations, and television, are examples of what is expected [33].
- Dynamic sources. Although these are transmitters that regularly broadcast in a manner that is not monitored by the internet of things’ system for such sources to yield energy, an intelligent WEH is required to continuously monitor the channel for potential harvesting opportunities. Unknown ambient sources include Wi-Fi access points, microwave radio links, and police radios, to name a few examples [33].

### 3.3. RF Energy Transfer between Mobile Devices

Wireless communication devices are able to serve as RF sources to transfer power among nearby devices stably. Previously, certain RF energy transmitters/receivers were designed to simultaneously transmit/receive power and information. The power splitting or time switching setting provides a cheap approach for sustainable wireless system operations in the absence of any transmitter modification. It allows a similar antenna or antenna array to be used for both the RF-EH and the information receiver. In short, mobile devices can be used to transfer information-based RF energy for the relay nodes to prevent the unbalanced consumption of energy [34].

## 4. RF Concepts and Principles

Using Maxwell’s equation concept to fully comprehend electromagnetic waves, it is possible to implement the physics that underlie microwave antennas [35]. Maxwell characterized the properties of magnetic fields and electricity in terms of electromagnetic waves and described the antenna functions for transmitting and receiving. Classical electromagnetism predicted that electromagnetic waves with infinite frequencies travel at the speed of light, which indicates the primary sign of the presence of the electromagnetic spectrum [36]. The existence of the electromagnetic spectrum was demonstrated by the behavior of a valence electron that simultaneously loses energy and shifts to a lower energy state from its previously higher energy state [37,38]. According to the principles of energy conservation, the electron radiates energy known as photon energy. The photon continues to travel indefinitely until it comes into contact with another electron in an atom along its path of movement. 

When the energy-carrying photon hits the electron, the electron may absorb the energy and become excited. During the photon process, the energized electron would move from the valence band to the conduction band. Once it transfers its energy, it disappears. Hence, the waves formed by the photons are described as electromagnetic waves, and they can exist in all forms of medium (liquid, gas, or solid). Interestingly, the RF energy is able to be transferred via EM [28]. The nature of the ambient RF energy overcomes the necessity for self-sustained. powered multipurpose electronic components by extracting nearby wireless RF energy sources, such as broadcast stations, cell phone towers, Wi-Fi hotspots, and RF emitting devices [39,40,41,42]. The constant, abundant supply of RF energy in nature provides an edge over other conventional energy sources, including thermal, solar, vibration, mechanical, and wind [43,44,45,46,47,48].

By implementing the above concept, microwave antennas are regarded as the essential element in RF-EH systems [49,50,51]. Whereas the RF-EH system is based on freely available RF energy in the environment, electromagnetic waves have different characteristics based on frequency, distance, and the conducting environment [52,53]. The design of the antenna should take into consideration the selection of potential traits based on the specifications of the application in perspective for the characterization of the electromagnetic waves. Determining the free space path loss, which defines power dissipation in space, requires knowledge of the transmitting wave frequency, gain, and distance between the transmitting and receiving antennas [54,55]. In order to accomplish RF-EH, antennas that can receive EM energy from the environment are used. These antennas are available to existing services that use ambient RF energy sources. There are two types of electromagnetic power sources: near-field and far-field.

### 4.1. Radio Frequency Energy Harvesting in the Near Field

Electromagnetic induction and magnetic resonance are two techniques that are commonly used in near-field applications to produce electric power and the ability to power devices that are within a wavelength of each other [56]. Ref. [57] explains an example of near-field RF-EH in which unintentional RF energy from 10 kHz to 1 GHz (wideband) was scavenged and converted to usable energy in a railway environment. A metamaterial with a wideband frequency of 350 MHz and a load of 8.5 Kohms was used to deliver up to 84% of the energy. 

### 4.2. Radio Frequency Energy Harvesting in the Far Field

Antennas can receive EM energy in the form of RF signals, which can travel for several kilometers in the RF-EH far-field before being transformed to power by rectifier circuits [53]. The ambient far-field RF-EH and the dedicated far-field RF-EH are the far-field RF-EH [57].

It is possible to charge a receiver’s battery via a circular disk transmission model using an EM wave transmitter Tr_s_ to transmit power to a receiver’s Rv_s_ located within its charging range, R_c_. The received power of omnidirectional antennas operating in free space varies inversely to d^2^_rt_, and it is assumed to be 0 when *d_rt_* > R_c_, where d_rt_ is the distance between Tr_s_ and Rv_s_. The obtained power is provided by:

(1) $$P_{r}=P_{t}G_{t}G_{r}{\left(\frac{\lambda }{4\pi d_{rt}}\right)}^{2\;}\;\;\;$$

where is the wavelength *λ*, *G_r_* is the sequential receiver gain, and *P_t_* *G_t_* is the power of the transmitted radio frequency signal multiplied by the linear transmitter gain. For lack of a better description, a transmitted power of 3 W will be received as 0.325 mW at a distance *d_rt_* of 5 m for 0.328 m at 915 MHz and *G_r_* = 3.98. The receiver converts the received power to a DC voltage and stores it in a capacitor after receiving the RF signal. As a result of the receiver, converter, and capacitor, a sensor equipped with these components can harvest energy and store it in a battery for later use. In practical terms, the amount of received power will be heavily influenced by the propagation properties of the environment [58]. A measure of power loss in space is free space path loss (FSPL), which is the loss of signal power during propagation in free space. For the far-field region, regarding the FSPL, PL is expressed as:

(2) $$P_{L}=\frac{P_{T}}{P_{R}}=\frac{{\left(4\pi R\right)}^{2}}{G_{T}G_{R}\lambda^{2}}=\frac{{\left(4\pi fR\right)}^{2}}{G_{T}G_{R}c^{2}}=\frac{4}{G_{T}G_{R}}{\left(kR\right)}^{2}$$

(3) $$P_{L}=20\log_{10}\left(f\right)+20\log_{10}\left(R\right)+20\log_{10}\frac{4\pi }{c}-G_{T}-G_{R\;}$$

where *f* is the frequency (MHz), *R* is the distance (km), and *G_T_* and *G_R_* are the gains (dBi). 

The Friis transmission and equivalent isotropically radiated power (EIRP) represent two crucial upper boundaries in the design of the RF-EH. Firstly, the available power at the antenna side should not ever go beyond the EIRP irrespective of the range. Secondly, when the power is determined at a distance, the power drops nearly below the power density of the Friis transmission except in distinctly reflective environments. The path loss is considered in the analysis to represent the power signal in the far-field region [59]. Although it is constantly available, radio waves pose a concern over the potential penetration of EMI/EMC through the human body, which the world health organization classifies as a potential risk [28,60]. Nevertheless, RF-EH systems remain a reliable, robust, and feasible solution to address the increasing demands for low-powered devices for domestic and commercial purposes [61,62,63,64]. 

## 5. Evaluation of Wireless Power Harvesting Metrics

The evaluation of multiple parameters is required to determine the performance of a WPH design. The evaluation merits vary depending on the type of application. Regardless, major parametric values, including sensitivity, efficiency, output power, and operation distance, form the standard evaluation criteria for comparative analysis. Nevertheless, trade-offs exist between the aforementioned parameters, such as the overall efficiency and operational distance. Besides these qualities, other additional manufacturing factors, such as cost-effective price, fabrication process maturity, and the feasibility for bulk manufacturing, are also essential parameters [28]. 

### 5.1. Efficiency of Power Conversion

The difference between the input RF power and the output DC power at the load is used to calculate the efficiency of a rectifier circuit. This is how it is mathematically demonstrated:

(4) $$\eta_{PCE}=\frac{P_{load}}{P_{retrieved}}$$

where *P_load_* represents the power supplied to the load, while *P_retrieved_* is the power harvested by the antenna. Note that the transmission loss of RF in space is not included in the expression. The circuit’s conversion efficiency strongly influences the rate at which energy can be captured by a rectifier circuit. The more efficient the circuit is, the better it is. Therefore, a near-100% efficiency is required, which means that all the energy that reaches the rectifier is converted to direct current energy. Impedance matching circuits must be carefully optimized, and the load resistance at the system’s output must be increased [65]. The following factors determine the efficiency of an RF-EH system:

- The efficiency and gain of the receiving antenna are the primary considerations in its design.
- The impedance matching ensures the maximum power transfer.
- The rectifier circuit’s power efficiency.

### 5.2. Sensitivity

The sensitivity is the measuring of the required minimum power to activate the system operation, which is expressed as follows:

(5) $$\mathrm{Sensitivity}\;\left(\mathrm{dBm}\right)=10\log_{10}\left(\frac{P}{1mW}\right)\;$$

where *P* represents the smallest amount of power required to complete a task by the system. Impedance matching networks are one approach to increase the sensitivity of the RF-to-DC rectifier circuit [66]. Improving sensitivity with regulation functions using a dc/dc converter leads to better adjustment in various RF energy harvesting applications [67]. The sensitivity, which is affected by the threshold voltage of the metal–oxide–semiconductor (CMOS) technology, is higher at the lower CMOS threshold but also causes more leakage current, leading to reduced overall efficiency [68].

### 5.3. Peak Passive Voltage

A bridge rectifier, a single diode rectifier, or an ideal diode rectifier can be used to generate the passive peak voltage. Diodes are used to remove the input signal’s negative voltages. A softening capacitor is connected to the rectifier circuits to decrease rectification ripples, resulting in a smooth DC output [69]. Defining the rectifier’s sensitivity and efficiency is the relationship of Vpeak to VTH at the input. Allowing Vpeak to fall below VTH disables the multiplier, identifying the sensitivity. The rectifier output voltage is also related to Vpeak, as the number of stages in the rectifier ladder determines the output voltage. Passive voltage boosting can be achieved by connecting the reactive and resistive components, as shown in Equation (6) [70].

(6) $$\frac{V_{rec}}{V_{ant}}=\frac{R_{rec}+jX_{rec}}{\left(R_{ant}+R_{rec}\right)+j\left(X_{ant}-X_{rec}\right)}$$

where:

*V_rec_*: the input voltage of the rectifier. 

*V_ant_*: the voltage source of the antenna.

*R_ant_*: the resistor of the antenna. 

*jX_ant_*: the inductor of the antenna.

### 5.4. Dropout Voltage Regulator

The dropout voltage regulator includes a linear voltage regulator, a switching voltage regulator, and a control logic. Voltage regulators, such as linear low dropout (LDO) regulators, can keep output voltages stable, regardless of supply voltage or load current fluctuations. The LDO linear regulators perform two functions: varying or regulating the output voltage of the load. The second must maintain the required output voltage despite variations in load current or power supply voltage. Concerning noise isolation, they outperform switching converters. Many portable battery-powered systems rely on the LDO regulator [71]. Because of the dropout voltage V_DO_ = (V_IN_ − V_OUT_), the power efficiency of LDO_s_ is fundamentally limited. As the dropout voltage rises, the power loss caused by resistive division becomes more severe. The regulation is performed in accordance with Equation (7).

Regulator Dropout Voltage = Vdropout = Vout − VDD(7)

## 6. Radio Frequency Environments 

Many telecommunications base stations (TBSs) and high-power digital television (DTV) transmitters in urban and suburban areas contribute to ambient RF energy. Suppose mobile devices, Wi-Fi access points, and home appliances such as microwave ovens are included. In that case, the density of these sources and the resulting ambient power density can vary significantly from one location to the next [72]. 

Due to the high power density in urban areas of DTV, GSM900, GSM1800, and 3G, these bands have been identified as potential harvesting bands. There is a greater chance to capture considerable electromagnetic energies in urban areas than in semiurban areas. The urban areas have more RF transmitters on top of buildings than semiurban areas [73]. Many empirical models have been developed to better understand the factors and phenomena that influence the power levels of electromagnetic signals during their propagation in urban areas [74]. The wide disparity in harvestable RF power between urban and semiurban areas and rural areas shows that the environment influences the amount of RF power that can be harvested. Rural areas have lower densities of base transceiver stations, user transmitting elements, and different morphologies, contributing to this discrepancy [75]. To filter the channels with the highest measurements in each band, a banded input RF power density threshold was chosen [26]. RF measurements in urban, and semiurban areas are summarized in Table 1.

## 7. RF-EH Circuit 

Although RF power is always present in the surrounding environment, the power density is generally low. In fact, regular RF power harvesting systems have small RF-to-DC PCE, notably when the harvested radio frequency power is limited. The small, converted energy is difficult to apply due to the presence of various losses and limitations in all parts of the RF power harvesting system. Hence, the design of each module must be well-developed to ensure the whole system becomes feasible [76]. A typical RF-EH circuit is composed of an antenna, an energy conversion module, a matching network, and a load. 

**Figure 2:**
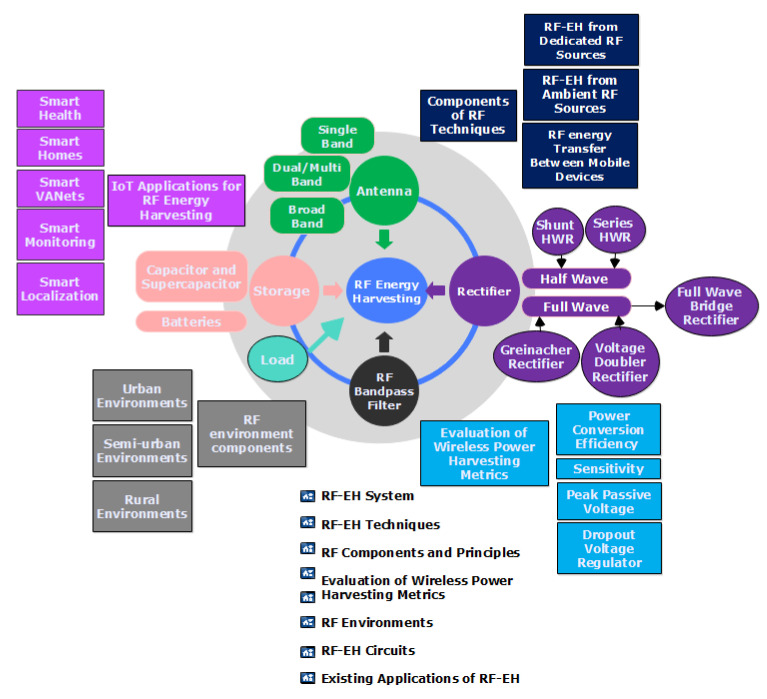
Taxonomy of literature on radio frequency energy harvesting.

A rectifier, RF input filter, antenna, and impedance matching network were all part of the rectenna system design. The RF energy is detected and received by the antenna, and then converted to a DC signal by the rectifier. The RF input filter, also known as the “pre-rectifier,” suppresses the harmonics generated by a nonlinear component in the rectifier. The power transfer is maximized by the matching network between the rectifier circuit and the input filter. Regardless of the application, the fundamental operation of a rectenna system is the same [77]. Based on the taxonomy shown in Figure 2, this study looked at the main parts and structure of research on radio frequency energy harvesting. 

### 7.1. Antenna Configurations

One of the most critical parts of a rectenna system’s front-end is the antenna. The antenna serves as the RF power receiver and converts them into a DC signal via other components of the rectenna system over the following stages. The effectiveness of the deployed antenna significantly influences the complexity, size, and performance of a rectenna system. The desirable properties of an antenna include a broad range of operating frequency, low-profile, omnidirectional radiation pattern, high gain, compact size, and circular polarization (CP) [77]. 

#### 7.1.1. Antennas Single Band

Single-band antennas in rectenna systems are designed to operate in a single narrow frequency band. Based on the perspective of performance, various parameters are taken into consideration during the design of the single-band antennas. The CP is a crucial parameter to improve the overall rectenna output power by achieving a steady power output from the antenna [78].

Furthermore, the CP antenna is a vital component to improve the overall efficiencies in rectenna systems, as it is able to receive RF energy with minimal loss in any plane. To date, a number of single-band antenna configurations have been investigated to achieve effective CPs. 

Moreover, the characteristic mode (CM) theory is applied to the design of polarization reconfigurable antennas that can switch between linear, left-hand circular, and right-hand circular polarization via electronic (RHCP). For example, the main radiator in [79] is considered a square patch with an I-shaped slot. It has two orthogonal CMs with the same modal significance and 90 characteristic angle differences at 2.4 GHz [79]. A new CP antenna design proposes a planar dipole array antenna and a center-fed Gysel power divider. The center-fed Gysel power divider fed two uniform linear array antennas with two planar dipole antennas, meandering the branch lines of the center-fed microstrip Gysel power divider, which allowed it to be reduced in size. It produced an excellent CP wave and a highly symmetric structure [80]. 

Patch antennas with inhomogeneous substrates are proposed in [81]. To control the inhomogeneity of the substrate, it is composed of ethyl acetate and air. Because of the patch antenna’s inhomogeneous substrate, excitation of two orthogonal fundamental TM modes with a 90° phase difference is simple, resulting in CP radiation. Finally, the CP can be reconfigured using the fluidic control method [81] by dynamically changing the substrate’s inhomogeneity. At 2.4 GHz band, a dual-port square patch antenna with polarization diversity (PD) is proposed in [82]. It is possible to generate linear polarization, left-handed CP (LHCP), or right-handed CP with the proper control of p-i-n diodes (RHCP). Dual linear polarized antennas can also be created in LP modes by obtaining two orthogonal waves simultaneously. Reconfigurability in both the patch antenna’s frequency and polarization are demonstrated. By employing 12 varactors with two independent voltages, a fractional bandwidth of around 40% can be achieved while allowing the user to select from linear (LP) and circular polarization (CP) [83]. 

A simple, low-cost, and efficient reconfigurable circularly polarized patch antenna array was demonstrated in [84] for the early detection of brain cancer. Measurements of S11 by single, double, triple, and quadruple antenna elements are used to detect the tumor [84].

In [85], a CP was generated using a semieccentric annular dielectric resonator antenna (DRA) and fractal-shaped DRA. CP can now be achieved using a new method for modifying the DRA geometry. For future 5G applications, a low-cost 16-port nonplanar multiple-input-multiple-output (MIMO) antenna system is introduced [86]. Biomedical applications will benefit from a CP ground radiation antenna with a wide AR bandwidth and low-profile properties. The antenna shown here is proposed to work in the ISM band (2.4–2.48 GHz). Coplanar waveguide feed (CPW) is asymmetric, allowing CP radiation to be realized [87]. Helical slot antenna CP radiation is examined in this letter.

High-gain omnidirectional coaxial line-feeding CP helix antennas with simple feeding structures based on the radiation of a slot antenna are presented. To improve CP performance, helix- and rectangle-shaped slots are cut into an outer conductor. In addition, a cone-shaped inner conductor is used to match impedance. The proposed antenna in [88] can be fed with a stepped structure instead of redundant feed structures to simplify the feeding of the CP antenna. An innovative CP-configurable liquid dielectric resonator antenna is being studied in this communication—the author in [89] proposed resonator antennas with circular polarization reconfigurability. The fluid dielectric used in this design is Ethyl Acetate (ԑr = 6.6), held in a 3D-printed container excited by one probe. Left and right zones are created in the container in order to implement the CP reconfiguration. A new circularly polarized (CP) origami antenna design has been proposed [90]. It is folded into an origami tetrahedron to serve as the antenna’s base. Triangular monopole elements perpendicular to each other make up the antenna’s structure. Both elements are excited with equal magnitudes and a 90° phase difference, whereas circular polarization characteristics can be achieved. A polarization-adjustable reconfigurable eccentric annular slot (EARS) antenna has been suggested. Two identical perturbation slots (PSs) are loaded beneath the eccentric ring-shaped slot to generate orthogonal circular polarization (CP) radiations. 

In order to switch between LHCP, RHCP, and LP, the proposed antenna incorporates a PIN diode switch across each PS [91]. However, an antenna that can be reconfigured by using gravity to control the self-circulation of liquid metal (LM) is explained in [92]. For reconfiguration, the patch is loaded onto the microstrip antenna with a polydimethylsiloxane (PDMS) structure, which has a square ring channel controlled by gravity [92]. Another unique design has been proposed in [93] with both a DGS-equipped and a nonequipped cylindrical slot antenna integrated with a circular cavity. The antenna’s DGS and non-DGS design results are compared extensively [93]. In contrast, Helical antennas are typical antennas with two primary modes of operation: normal and axial. Helix axial mode radiation is most substantial in the direction of the helix axis [94]. 

Rectennas for energy harvesting, such as directional ones [95,96], circularly polarized ones [97], and dipole ones [98], are currently being developed in large numbers. To harvest radio frequency energy in the GSM-1800 band, [99] has developed a multiport pixel rectenna comprised of a triple-port pixel antenna optimized for use with a triple-port rectifier. Furthermore, 19% of the efficiency can be achieved when the input radio frequency power is at −20 dBm. A global rectenna device with an RF to DC conversion efficiency ranging from 16.3 to 45.3%, depending on the amount of harvested power, is created by synthesizing two-port grid-array (TWR) antennas at 2.45 GHz with a coplanar, strip-line-based rectifier, as demonstrated in [100]. Rectenna synthesizers operating at 2.45 GHz can be created using a new four-cascaded element array of orthogonal patches [101]. The rectenna’s maximum RF to DC efficiency is 77.2%. Therefore, the use of a single-band, high-gain antenna suits well in the design of the rectenna. The techniques that reported the increase in the gain [102,103,104,105,106,107,108] also increased the rectenna’s dimension or the design’s complexity. Masius et al. [109] reported that a multilayered structure was the most preferable technique to obtain high gain. In order to broaden the application of the rectenna, the antenna size must be small, followed by the rectenna size. 

#### 7.1.2. Broadband/Wideband Antennas 

Broadband and wideband antennas are designed to harvest energy from various sources over a wide frequency range. The study on broadband antennas has been reported in several works of literature. In [110], the authors show how a flower-shaped slot in a cross-dipole antenna can improve impedance matching between 1.8 GHz and 2.5 GHz. A compact and wideband slotted antenna at the LTE band was demonstrated by reference [111] and performed well with a wide bandwidth of 2 GHz to 3.1 GHz. Even with wide band antennas, it is difficult to cover all the communication band channels dispersed distribution in both the ISM and communication bands. Author [112] was able to achieve broad wide-angle ambient wireless energy harvesting (WEH) and directional wireless power transfer (WPT) for 2G, 3G, 4G, and 5G communication bands and ISM-2.4 GHz and ISM-5.1 using a novel back-to-back microstrip antenna. The first textile-based antenna to cover the fifth generation (5G) spectrum is shown for wearable applications as a broadband textile antenna [113]. The author in [114] demonstrated the compact wideband rectenna using an inverted L open circuit stub for the matching network (MN) and a voltage double for the rectifying circuit with a minimal footprint size (58 × 55 mm^2^). In addition to WiMAX signals, the proposed rectenna can use transmissions from wireless applications such as WLAN 802.11a, UMTS 2100, GSM 1800, band g systems, and WiMAX up to 28% for power conversion. S. Agrawal et al. [115] combined an underlying narrow rectangular notch slot in the ground plane and the DRA to widen the antenna bandwidth (BW). Millimeter-wave applications are being addressed by a single-layered multiple-input-multiple-output (MIMO) antenna operating at 28 GHz and loaded with compact planar-patterned metamaterial (MTM) structures [116].

#### 7.1.3. Antennas with Multiple Bands 

The multiband antenna exhibits a wide range of advantageous frequencies suitable for various situations once used in rectenna applications. Even though the antenna receives more power from the surrounding environment when using this method, it is less efficient than the previous method. One of the most difficult challenges for researchers working in the field of radio frequency energy harvesting is the low RF-to-direct-current conversion rate that is necessitated by the low available RF power density. The antenna arrays are ideal in terms of output power because they have a large output area. On the other hand, they are difficult to integrate and take up a significant amount of chip space. In order to increase output power, it may be preferable to use a system that has three or more band frequencies. Consequently, the development of compact multiband circularly polarized rectennas for environmental RF energy harvesting is required [117]. A compact multiband rectenna for WLAN, Wi-MAX, GSM, and satellite communication bands is demonstrated in [117]. The rectenna system is fractal in geometry and has six radiating bands: GSM 1.8 GHz (1.6–2.1 GHz), GSM 0.9 GHz (0.8–1.2 GHz), Wi-MAX 3.5 GHz (3.1–4.0 GHz), WLAN 2.5 GHz (2.2–2.8 GHz), WLAN 5.5 GHz (5.3–6.4 GHz), and 7.35 GHz (7.0–7.8 GHz). 

In [118], the author describes a dual-band rectifying antenna that can be reconfigured for 5.2 and 5.8 GHz rectification. Using an input power of 15 dBm, the proposed rectenna can achieve maximum conversion efficiency (CE) of 65.2% at 4.9 GHz and 64.8% at 5.9 GHz. The presented design is an example of novel ultrawideband energy harvesters in the UHF and low microwave spectrum with octave and decade bandwidths; this is accomplished by optimizing the dimensions of a broadband impedance matching nonuniform transmission line (TL) [119]. The dual-band slot antenna proposed in [120] has a novel symmetrical structure and is small in size. Antenna feeding is accomplished via a coaxial cable with a structure composed of two metal plates. The printed slot antenna structure consists of a single metal surface on a FR-4 substrate with a thickness of 1.6 mm. In terms of WLAN/WiMAX applications, this slot antenna is ideal [121]. 

To address the dual-band omnidirectional antenna’s lower gain and bandwidth, as well as the challenge of low efficiency, a new CRLH dual-band rectenna has been demonstrated [122]. For the two ISM bands (2.4 and 5.5 GHz), the authors in [123] propose a dual-band microstrip antenna driven by differentially driven microstrip antennas. The proposed dual-band antenna has a peak efficiency of about 90% in either band or is constructed with low-loss materials. In the GSM900 band (890–960 MHz), the antenna has an 8.5 dBi gain. Then. There is the 5.8 GHz antenna, which is compact and has a 10.1 dBi gain [124]. 

In [125], a circular patch antenna (CPA) with a gain of 8.3 dBi and 7.8 dBi was created to cover the frequency bands of 1.95 GHz and 2.45 GHz. In reference [126], a tree-like receiving antenna with a gain of 2.16 dBi and 1.92 dBi was introduced to operate in the 2.45 GHz Wi-Fi and 3.5 GHz WiMAX bands. Log-Periodic Dipole Array antennas are triangular-shaped dipole antennas [127]. A 3.5 GHz and 5.8 GHz dual-band antenna has been developed and optimized. To maximize the antenna’s gain, a genetic-algorithm-optimized rectangular unit cell has been etched on the patch radiating element. A receiving antenna collects energy from two high-gain bands [128].

A high-gain multiband antenna is able to increase the receiving power of the antenna. Various techniques have been reported, such as the air gap technique [129], differential-feeding [130], the aperture-coupled feeding technique [131], and the antenna array technique [132,133], and are affected either by the large antenna size or design complexity. In contrast, a small antenna size used in a multiband rectenna enables the increase of the rectenna applications. Varying techniques have been adopted to compress the antenna size, such as the meandered lines concept [134], fractal geometry [135], the multibending curves method [136], and the Sierpinski fractal technique [137].

### 7.2. A rectifier’s Configuration

Signal rectifying can be performed with diodes, transistors, and complementary metal–oxide–semiconductor (CMOS) technology. A rectifier is a device that converts alternating current (AC) created in an antenna by received radio frequency signals into direct current. Signal rectification topologies are classified as follows:

1. Half-wave (HW) rectification is either the positive or negative half of an alternating current wave passing while the other half is blocked. Rectifiers produce unidirectional but pulsating direct current (DC).

2. Full-wave (FW) rectification is a technique that converts the entire input waveform to an output waveform with constant polarity (either positive or negative) [138]. 

Completely rectifying an input waveform converts both polarities of the waveform to pulsing direct current (DC) and results in a higher average value output voltage.

**Figure 3:**
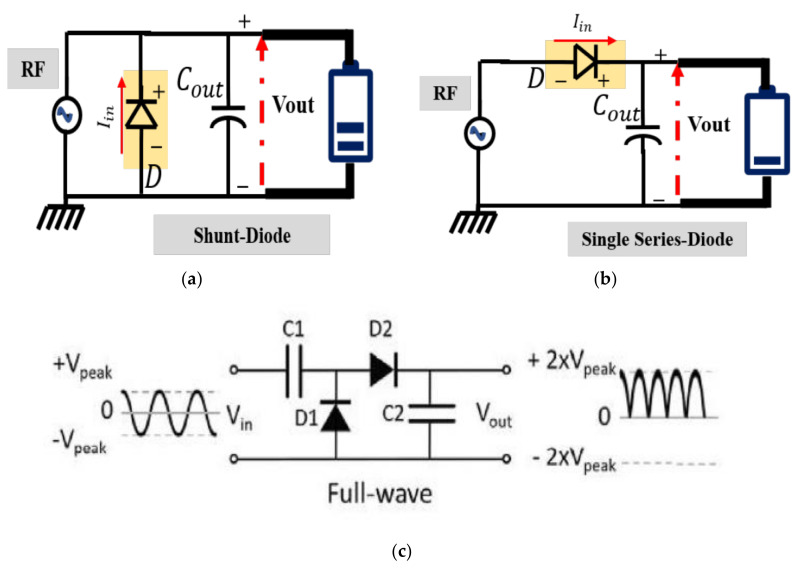
Half-wave rectifier with (**a**) shunt configuration and (**b**) series configuration [140], and (**c**) full-wave rectifier configuration [16].

Half-wave rectifiers produce far more ripples than full-wave rectifiers, necessitating the use of far more filtering to remove alternating current frequency harmonics from the output. The most common rectifying circuit consists of a single diode in a serial design that functions as a half-wave rectifier (HWR). Lower built-in voltage diodes, such as Schottky diodes [138], can generally achieve higher conversion efficiency than higher built-in voltage diodes. The series and shunt single diode topologies provide the option of reducing the loss of the diode, which is measured in terms of the diode junction resistance [139]. Figure 3 shows the half-wave rectifier in three different configurations: (a) shunt, (b) series [140], and (c) full-wave rectifier [16]. 

Schottky diodes have been utilized, and the Dickson charge pump is the most frequent topology [141]. However, due to the high threshold voltage of diodes, obtaining an efficient output is difficult. Metal–oxide–semiconductor field-effect transistor (MOSFET) technology has overcome the limitations of diodes and has emerged as a viable replacement for rectifiers [142]. The voltage developed at the output is given as follows:

(8) $$Vout\;\left(dc\right)=2Vin\;\left(RF\right)\;-\;Vth$$

It is necessary to use four diodes in an FW bridge rectifier design to reduce the number of diodes and hence minimize the total losses. Given that the resultant output may be decreased by accounting for the losses associated with the diodes, a full-wave rectifier was discovered to be a suitable alternative for higher input power-density levels. When the power received is insufficient to power the circuit, the rectifier input must be magnified to provide enough power. Alternatively, a rectifier circuit with a multiplier may be employed in certain situations [143]. Circuit designs for rectifiers can be divided into three categories: the primary rectifier, which uses a capacitor and single diode; the voltage doubler, which uses capacitors and two diodes; and the voltage multiplier [138].

**Figure 4:**
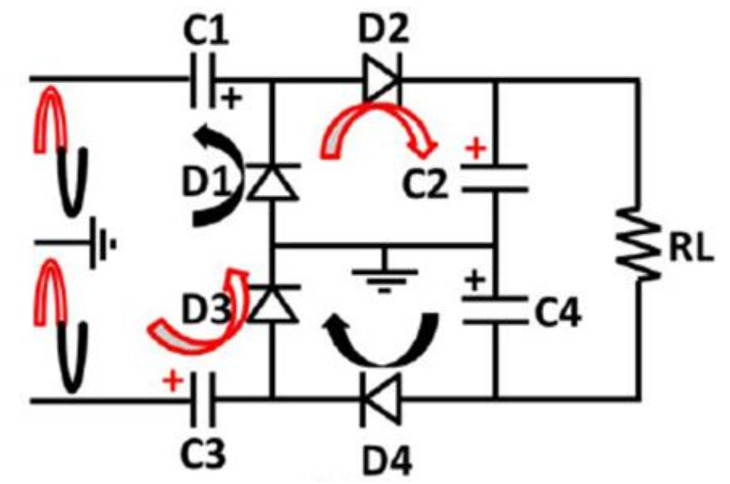
Greinacher rectifier configuration [144].

The performance of the rectenna is further improved by the use of a Greinacher rectifier circuit (GRC) [144,145]. GRCs are analogous to two-stage VDR circuits arranged in a bridge configuration, as illustrated in Figure 4. It consists of two branches, each with a pair of diodes, and it is connected to a power source. In addition, each diode’s biasing voltage can be moderately generated by the output of the previous diode, reducing the need for external power. As a result, the proposed GCR design is suitable for a wide operating frequency range (1.8–2.5 GHz) [144], while the proposed GRC’s 180° phase difference was achieved using a hybridized rat-race coupler [145].

### 7.3. Topologies of the Impedance Matching Network (IMN) and the Input RF Filter

#### 7.3.1. RF Filter Designs

The rectifying circuit (rectenna) filters the input RF signals to prevent harmonics from being generated by the rectifying circuit’s diode. Input RF filters are used in a stacked setup to allow required frequencies to pass through a broadband or multiband rectenna. Few studies have examined the input RF filters used in the rectenna design, and those that have done so have been published. A low-pass filter with two additional band-stop filters added to it was found to be effective in preventing second-order harmonics of both frequencies [146].

#### 7.3.2. IMN Configurations 

It is critical to match impedances in RF circuitry because it is necessary to do so in order to avoid standing waves and to ensure efficient power transfer from the source to the load.

##### Single-Band Matching Network

The antenna and the rectifier circuit are connected by the impedance matching network (IMN), which transfers optimal power from the antenna to the rectifier circuit. IMN topologies have been studied in a variety of ways so far. S. Agrawal et al. [147] compared the rectenna performance with and without a matching network (MN), where the rectenna with an MN demonstrated better results. The π-type MN performance was higher compared to the L-type MN. In addition, H. A. Sodano et al. [148] employed an L-section network to obtain suitable impedance matching. Although the proper impedance matching (IM) is easier to achieve for a single-band rectenna, the proper impedance matching is much more challenging to achieve for both a multiband and a broadband rectenna. 

##### Multiband Matching Network 

Recently, the author in [149] employed a dual-band multisection MN comprising the configured rectifier circuit. A stack of rectennas with triple dual-band bandpass IMNs on each rectenna and a related rectifier circuit was developed [128]. In addition, a fourth order dual-band IMN was applied in [150] via a combined series and parallel LC pair. Meanwhile, a three-band IMN was employed using a couple of stubs [151]. 

C.-Y. Hsu et al. [152] implemented a quad-band MN with a series of MNs, where each MN was tuned to a specific resonant frequency. M. Zeng et al. [153] studied the operations of independently associated common matching (CM) and two-branch MNs with a voltage octuple rectifier. For the CM method, the MN was linked between the two-stage rectifier circuit and the input port. In contrast, the TBM methods were carried out by dividing the total feedline impedance of 50 Ω equally into two branches of 100 Ω. Therefore, the two-stage rectifier circuit exhibits a matching impedance of 100 Ω. The TBM technique obtained better impedance matching than that of the CM technique. Additionally, the rectenna performance with a two-branch MN also showed higher results than a standard MN. The rectenna size increased with an input RF filter, hindering their applications. Moreover, the holding harmonic rejection property of the rectenna suitably fitted the design. Interestingly, the rectenna itself holds the harmonic rejection property [154]. 

### 7.4. Power Management Module

A power management unit (PMU) ensures that the load receives appropriate power conditions based on the energy harvested. For instance, if the load necessitates a regulated or precise voltage, or if the load necessitates precise running times, a commercial or tailored power management system can be used, depending on the design constraints. Finally, supercapacitors are preferred in EH Wi-Fi systems as a result of their high power density, high loading and unloading speed, long-life cycle, small size, and low cost compared to batteries [155]. The primary goals of the PMU are to maximize the amount of energy transferred from the antenna to the reservoir in real-time and regulate the sensor node’s supply voltage using the harvested energy properly and efficiently [156]. To investigate the performance of different integrated designs of low energy harvesting, power management technologies, and energy storage, the majority of studies have relied on theoretical and lab experiment research methods [157].

### 7.5. Storage Element

To put it another way, “energy storage” refers to any technology that can convert energy from one form (such as electrical energy) into another form (such as chemical energy) (e.g., electrochemical). After storing the energy, it can be converted back into an immediately usable form. There are several different types of energy storage available in terms of capacity, power, and charging/discharging rates. Application requirements dictate the selection of a particular technology. Energy storage units must meet a specific set of environmental monitoring requirements, including small size, adequate capacity, and low environmental impact [158].

The energy harvesting circuit would continuously supply energy to the load. Using an energy storage device is not necessary for this situation. An energy storage device, on the other hand, is used to store the power that has been collected. Several parameters influence the impedance of the EHC, including the load impedance, input power level, rectifier, voltage multiplier topologies, and operating frequency [159].

The impedance of the load must be perfectly matched to the impedance of the circuit for optimal power transfer. As a result of this consideration, the optimal load impedance is fixed for each operating frequency and input power level. The energy harvesting circuit should be configured with the optimal load to achieve the highest PCE. Energy storage devices are considered part of the load [160].

#### 7.5.1. Capacitor and Supercapacitor

Electromagnetic energy harvesting (EMEH) uses capacitors and supercapacitors as storage devices. For storage, the EMEH system includes a capacitor [161]. The capacitor has the advantages of being readily available and being inexpensive. Although the capacitor has some drawbacks, the EMEH system does not use it because of its low power density and extended charging and discharging times. The supercapacitor has been shown to have lower internal impedance and longer lifetimes (measured in terms of charging and discharging cycles) [162].

Supercapacitors, on the other hand, can calculate the amount of residual energy with precision, taking into account charging and discharging capacity as well as energy density states (i.e., upper or lower). The disadvantage of the supercapacitor is the higher cost per kilowatt-hour. Using batteries to power autonomous sensors in remote areas has a number of drawbacks. Size, storage capacity, and lifecycle are just a few of the disadvantages [163].

#### 7.5.2. Batteries

Besides supercapacitors, batteries can be used as an alternative option to store energy or supply rechargeable energy in energy harvesting circuits. A variety of batteries are currently available, including Lithium–Ion (Li-ion), Lithium (Li), Sealed Lead Acid (SLA), Nickel Metal Hydride (NiMH), Nickel Cadmium (NiCd), and Nickel Metal Hydride (NiMH). Li and lithium alloy batteries have the edge over other batteries due to their high efficiency. The same battery model could be configured as a series connection of an internal resistance and an ideal voltage source [164]. As with supercapacitors, batteries are treated as part of the load in the energy harvesting circuit. Their charging efficiency varies as a function of the amount of current applied to the batteries during charging. In order to achieve maximum battery charging efficiency, it was necessary to identify the optimal charging current [165]. 

Contrary to supercapacitors and conventional capacitors, batteries have the advantage of exhibiting higher energy density. Nevertheless, the downside of batteries is their low power density and low lifespan compared to supercapacitors. A novel energy storage device, known as the supercapattery, was recently introduced. The electrode in supercapatteries combines the most effective components in supercapacitors and batteries, carbon nanotubes, and redox materials [166]. 

### 7.6. The Influence of Multiband Frequencies on the RF Harvester Architecture

**Figure 5:**
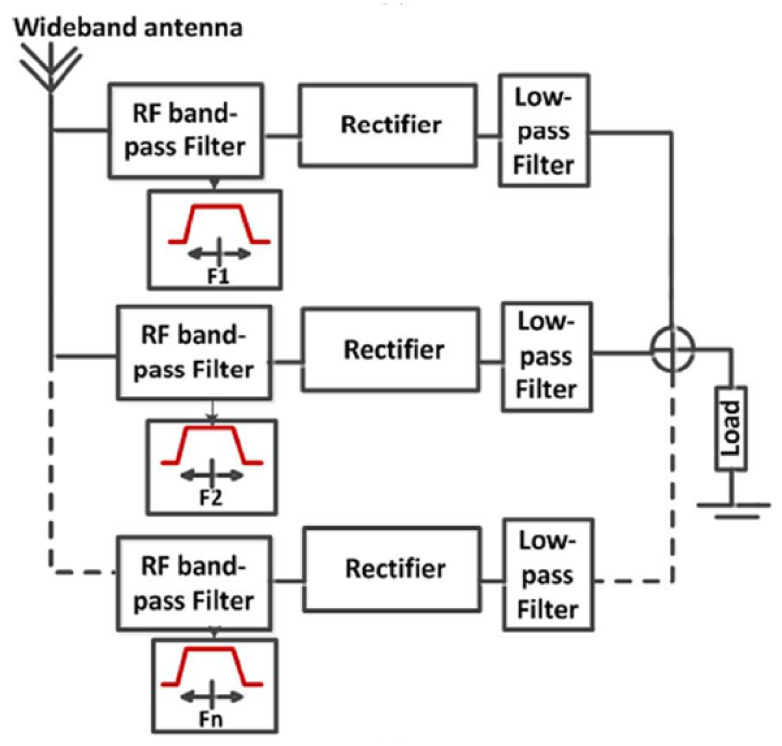
The schematic diagram of the architecture of the multiple RF circuits [169].

**Figure 6:**
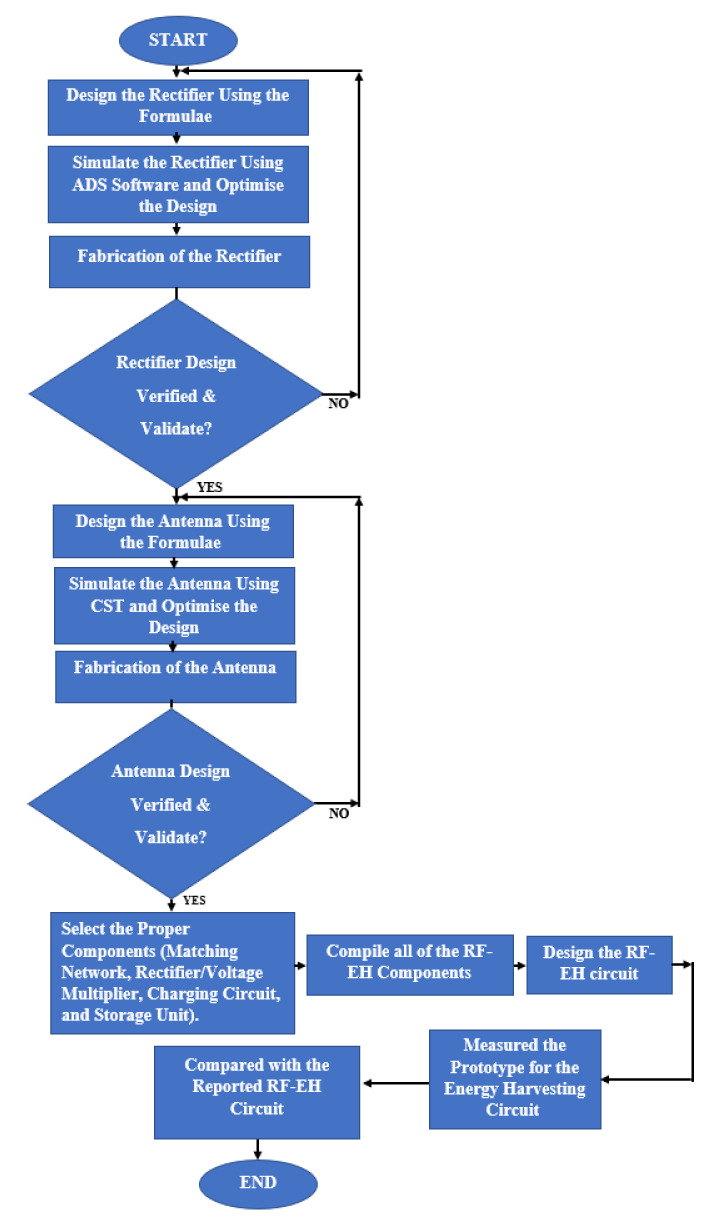
Methodology steps to design the radio frequency energy harvesting circuit.

Radio-frequency energy harvesting has received a great deal of attention in the research community over the last few decades. Although a single narrow-band design promotes high efficiency, the amount of DC output power is limited. A multiband, broadband, or rectenna array can accumulate more power from weak ambient sources and produce more output power than a narrow-band rectenna, but the trade-offs may include decreased overall efficiency and increased dimension. The main challenge of wireless energy harvesting at the moment is how to improve power conversion efficiency at low-input power levels across a wide frequency band [167]. Due to the wide frequency range of the ambient wireless signals (ranging from 200 MHz to 4 GHz), multiband and broadband rectennas may be a promising solution to these problems. Rectennas of this type can receive more energy from a lot of different channels and sources at the same time, which leads to more DC output power [168]. To harvest energy from multiple RF bands, several RF harvester topologies can be investigated. The main distinction is in the RF bandpass filter design. The filter’s function is to match the antenna impedance and the conjugate impedance of the rectifier input. Furthermore, the impedance of the rectifier’s input varies with frequency and incident power. The impedance of the antenna can also change depending on the frequency. As a result, adjusting impedances at a single frequency is easier than adjusting them across an entire RF band. Adaptation to multiple RF bands, according to this, results in two types of losses: those caused by impedance mismatch and those caused by filter complexity [169]. In situations where there are multiple RF energy sources that are easily accessible, the quantity of energy harvested can be increased, which is especially useful if the system is intended to operate over a wide frequency spectrum. Figure 5 shows the schematic diagram of the architecture of the multiple RF circuits [169]. The author in ref. [170] described the multiband, differentially fed rectenna as being very useful for RF energy harvesting applications because it would have all the potential benefits of a differential structure, such as poor common mode noise, proper harmonic suppression, and so on. It would also have other advantages, such as steadily increasing RF to DC efficiency at even low RF power levels, because it would be extracting from multiple sources at the same time. It is mostly because of this that a differential rectenna can work in three different frequency ranges. It will be more difficult to maintain the next generation of sensors and circuits because they will be built right into the products themselves. If you cannot obtain a replacement battery, then batteryless devices are your only option. The environmental impact of these distributed and ubiquitous electronics must be minimized in order to avoid increasing pollution risks and lowering human life quality, as well as conforming to the host object’s shape and cost [171]. Author in ref. [172] introduces a triple-band, high-gain multibeam ambient RF energy harvesting system for downlink channels of GSM-1800 (1.805–1.88 GHz), UMTS-2100 (2.11–2.17 GHz), and Wi-Fi (2.4–2.48 GHz) systems to overcome the challenge of battery charging and replacement in the IoT. Compared to previous work, this energy harvesting system provides a higher output DC power with a better RF-to-DC efficiency. As a result of its high antenna gain, this energy harvesting system is able to capture more energy from its surroundings while simultaneously taking advantage of frequency, spatial diversity, and polarization diversity. It is possible to change the methodology depending on what the researchers want to accomplish, such as using a single methodology (using only one method, software, and equation) or a hybrid methodology (combining several methods, software, and equations) (using multimethods, software, and equations). CST is used to design a radio frequency energy harvesting circuit, as shown in Figure 6.

## 8. Existing Applications of RF-EH

In this section, various rectennas were compared based on their size, operating frequency, substrate, and diode technology. Previously, a CP graphene field effect transistor (GFET)-based rectenna coupled with a monopole antenna was created to detect high-frequency RF signals [173]. A miniaturized, printed rectenna was also designed to harvest energy from ambient RF power around 2.45 GHz [174]. Meanwhile, a wearable rectenna array was introduced in which each rectenna uses a stack topology to minimize the element dimensions in the array. Cordura textile material was used to fabricate the design, which was durable, light, tough, and comfortable, making it suitable for wearable antenna design [175]. Furthermore, a transparent ultrawideband (UWB) antenna was proposed with a unique wideband rectifying circuit. The device aimed to harvest and convert EM energy from most telecommunication bands into electrical energy [176]. 

Additionally, the design analysis was carried out in [177] on a wideband rectenna. The study revealed that the combined antenna and rectifier supplied energy to low-powered devices, non-battery-powered sensors, and various IoT devices. Recently, a monopole wideband antenna array fed by a microstrip-to-slot-line transition feeding network was demonstrated. The feeding network was in reference to the slot-line stubs and radial microstrip to apply the microstrip-to-slot-line broadband transitions [178]. A unified, cloud-based IoT system was introduced for smart home applications [179]. 

**Figure 7:**
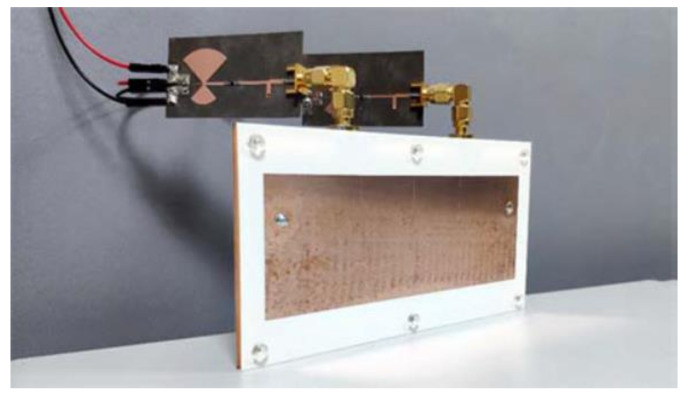
Photographs of the fabricated Rectenna [183].

A new rectifying-antenna design was introduced with an expanded power input range operating at 2.4 GHz for ambient (WEH) systems and wireless power transmission (WPT), as depicted in Figure 7 [180]. 

A highly efficient rectifier with an input power range for EH and a wide bandwidth was also suggested [181]. In terms of compact size, low cost, and a higher voltage output provided by the realized rectifier, which was optimized for low-input power conditions, a rectenna to harvest RF energy at the ISM band of 2.4 GHz was also designed [182]. A varying topology for the rectifier, in addition to the microstrip patch antennas, was employed to terminate the power splitter at the input and a voltage adder at the output to reduce the ohmic loss and leakage. 

**Figure 8:**
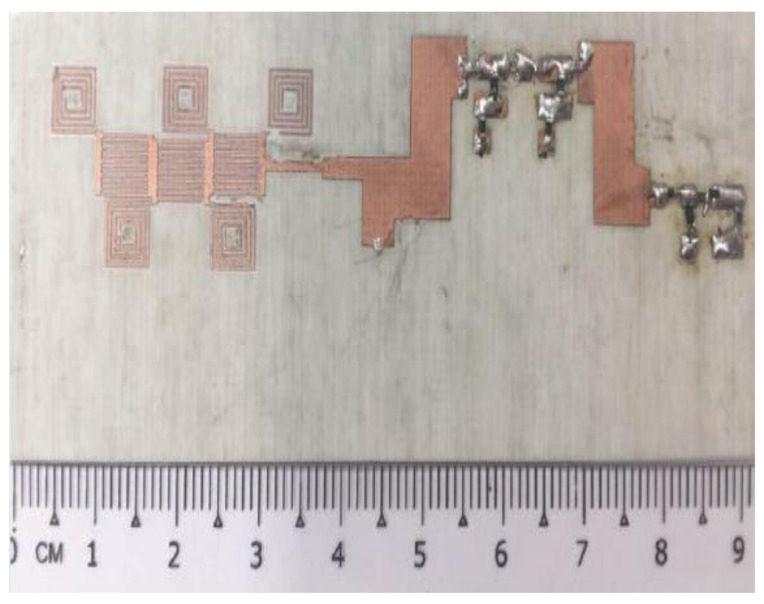
Fabricated prototype of the suggested rectenna [184].

In an RF-EH device, a short traveling wave series-fed patch (SFP) was used to improve the angle range. In contrast, a design based on the periodic circuit model, which included series-coupled resonators to aid the short series-fed patch design, was introduced [183]. Besides, K. T. Chandrasekaran et al. [184] designed a novel dual-band Composite Right/Left-Handed (CRLH)-based rectenna to address the poor efficiency and the poor gain bandwidth in the dual-band omnidirectional antenna, as shown in Figure 8.

T. S. Almoneef et al. [185] eliminated the use of an MN between the rectifier and the antenna by proposing a new approach to design the rectenna system. The ground plane supports the novel rectenna design, which consists of eight octagonal discs with a broad impedance bandwidth sandwiched between two varying layers on both sides of a single substrate. A Schottky diode was also placed on each layer next to the feeding location without an MN, resulting in a two-rectenna array. Furthermore, in ref. [186], the Huygens dipole rectenna has exceptional physical and radiation properties. Incorporated on a single Rogers 5880 substrate was a high-efficiency rectifier circuit and an ultrathin and electrically small Huygens dipole antenna (HDA). M. Wagih et al. [187] demonstrated a textile-based broadband rectenna for a fifth generation (5G) RF-EH and Wireless Power Transfer, which uses a novel wearable antenna design and a highly sensitive broadband rectifier. 

**Figure 9:**
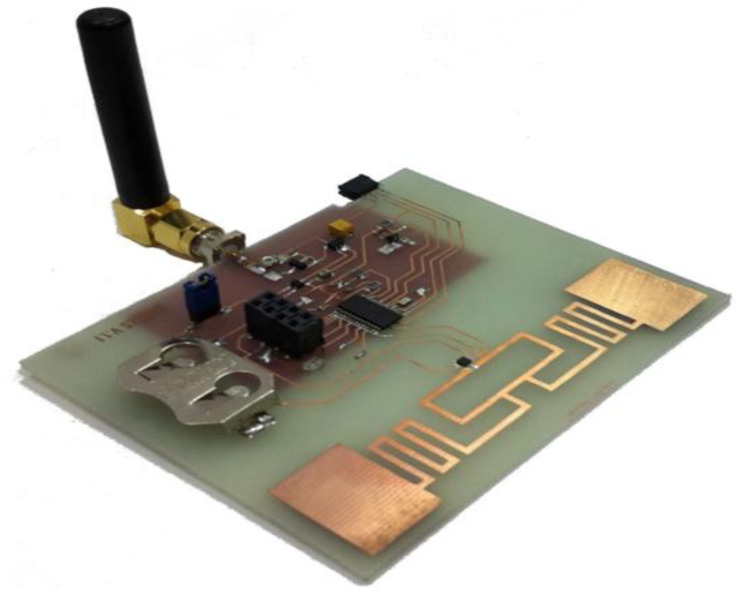
RAMSES Prototype [190].

An RF-EH and storage module for wearable applications can harvest 8.4 mJ of energy in less than four minutes from 915 MHz industrial, scientific medical sources [188]. A textile dual-band, dual-mode antenna was introduced for simultaneous wireless information and power transfer (SWIPT) applications, which was considered the first multiband microstrip antenna with an MN-less codesigned rectenna RF-EH application [189]. As shown in Figure 9, the radio frequency identification (RFID) augmented module for smart environmental sensing (RAMSES) was first introduced in [190] as a fully passive device with sensing and computation capabilities to investigate novel and unconventional RFID applications. Table 2 lists the comparative study on the variation of existing circuits for the RF-EH approach. 

Besides, as examined and verified in [191], the metamaterial absorber (MA) can be used for energy harvesting with a large fractional bandwidth (FBW). Researchers compared several substrate materials and fabrication procedures to examine micromachined antenna performance at 5 GHz for radio frequency energy harvesting (RF-EH) applications [192], as well as microwave applications [193,194,195].

## 9. Conclusions

This paper summarized the latest update on the advanced RF-EH technology. The paper presented a general outline of RF-EHNs, specifically the design, methods, and current applications. The study also reviewed the background issues regarding the circuit design and its implementations. A wide range of design issues associated with the resource allocation in RF-EHNs has also been addressed. The practical challenges and future directions in RF-EH techniques were also given. This novel technology would play a crucial part in replacing batteries in the foreseeable future. The abundantly available RF electromagnetic waves in the environment are harmless to the human body since they are able to penetrate through the soft tissues without inflicting any damage. Despite the progressive achievements over the years, various improvements remain to be further optimized to enhance the RF-Eh technology, including increasing the operational range, minimizing the transmission loss, optimizing the PCE, and reducing the system dimensions. In short, future studies should be focused on developing antennas with the capability to operate across multiple frequencies comprising 2.3 GHz (Wi-MAX), 2.4 GHz (WLAN), 2.6 GHz (LTE/4G), as well as 5.2 GHz (WLAN). Further studies should also consider improving the rectenna’s direct current (DC) voltage to provide the optimum transfer of power.

## Figures and Tables

**Table 1:** Summarizes the RF measurements in urban, semiurban, and rural areas [26].

Environment	Band	Number of Stations	Maximum PIN, dBm	S_BA_ Threshold nW/cm^2^
Urban	GSM-900	8	−19.2	230
	GSM-1800	7	−31.3	450
	DTV (during switch over)	10	−34	40
Semi-urban	GSM-900	2	−22.3	230
	GSM-1800	3	−43.5	450
	DTV (during switch over)	0	−37	40

**Table 2:** The comparative study on the variation of existing circuits for the RF-EH approach.

Ref.	Frequency (GHz)	Max Conversion Efficiency (%)	Circuit Size (mm^3^)	P_in_ (dBm)	Max Gain (dBi)	Max Harvested DC Output Voltage (v)	Substrate	Distance (m)	Diode Type
[173]	24	80	40 ×40 ×1.6	4.9	7.8	6.82	FR-4	1.5	Schottky
									CMOS
[174]	2.45	20	24.9 × 8.6 × 1.6	−20	0.8	0.097	FR-4	0.9	HSMS-2852 Schottky
[175]	2.45	-	160 × 130 × 0.55	–40 to 0	5	1.05@1.5 m	Cordura fabric	1.5	HSMS-2862
						1@2 m		2	Schottky
[176]	3.1–8	69	6.3 × 13 × 0.8	−10	3.2	-	FR-4	0.5	SMS 7630
[177]	1.975–4.744	88.58	40 × 45 × 1.6	0	4.3	10.703	FR-4	2	HSMS 270B
									Schottky
[178]	0.91–2.55	68	165 × 165 × 0.8	−10	5 to 8.3	0.243	FR-4	-	HSMS-285C
[179]	1.7–3	60	178 × 148 × 0.813	-	9.902	~3.7	Roger	0.75	SMS7630
							RO4003C		
[180]	2.4	50	63.7 × 45.6 × 1.6	−10 to 17	5.3	3	FR-4	1–2.5	HSMS 2850 and SMS7630
[181]	2.1 & 3.3	76.3	31 × 18 × 1	4 to 16	-	-	F4B	-	HSMS286
[182]	2.4	69.3	4 × 11.7 × 1.6	5.2	5.9	3.5	RO4003C	-	SMS7630
[183]	2.45	19.5–44.6	150 × 80 × 4	−9.48	8.53	-	RO4003	-	SMS7630
[184]	2.45 and 3.6 GHz	59%@	44 × 24.5 × 0.06	2	2.6 d@ 2.45 1.6 d@ 3.6	-	Rogers R04003	0.65	SMS-7630
		2.45							
		41% @3.6							
[185]	2.2	50	71 × 71 × 1.6	29	7:46	0.516 in parallel 1.087 in series	RT/duroid	1	SMS7621
							5880 Rogers		
[186]	0.909	88	99.5 × 26 × 0.508	−10	4.6	7	Rogers 5880	1.2	HSMS286C
[187]	20–26.5	70	32.6 × 16 × 4	27	8	6.5	Textile	0.12	SMS7630
									MA4E-1319
[188]	0.915	80	115 × 15 × 1.4	−7	2.3	1.8	Textile	4.2	BAT15-04R
[189]	2.4	63	-	−10	1.7	0.65	Felt	0.89	SMS7630-079lf
	0.83								
